# The battle of the sexes starts in the oviduct: modulation of oviductal transcriptome by X and Y-bearing spermatozoa

**DOI:** 10.1186/1471-2164-15-293

**Published:** 2014-05-21

**Authors:** Carmen Almiñana, Ignacio Caballero, Paul Roy Heath, Saeedeh Maleki-Dizaji, Inmaculada Parrilla, Cristina Cuello, Maria Antonia Gil, Jose Luis Vazquez, Juan Maria Vazquez, Jordi Roca, Emilio Arsenio Martinez, William Vincent Holt, Alireza Fazeli

**Affiliations:** Academic Unit of Reproductive and Developmental Medicine, Department of Human metabolism, The University of Sheffield, Level 4, The Jessop Wing, Tree Root Walk, Sheffield, S10 2SF UK; Department of Medicine and Animal Surgery, Veterinary Faculty, Regional Campus of International Excellence, University of Murcia, Spain, Espinardo 300071, E-30071 Murcia, Murcia, Spain; Sheffield Institute for Translational Neuroscience (SITraN), Sheffield, United Kingdom; Department of Computer Science, University of Sheffield, Sheffield, UK

**Keywords:** X and Y-chromosome bearing spermatozoa, Oviduct, Transcriptome, Sex selection

## Abstract

**Background:**

Sex allocation of offspring in mammals is usually considered as a matter of chance, being dependent on whether an X- or a Y-chromosome-bearing spermatozoon reaches the oocyte first. Here we investigated the alternative possibility, namely that the oviducts can recognise X- and Y- spermatozoa, and may thus be able to bias the offspring sex ratio.

**Results:**

By introducing X- or Y-sperm populations into the two separate oviducts of single female pigs using bilateral laparoscopic insemination we found that the spermatozoa did indeed elicit sex-specific transcriptomic responses. Microarray analysis revealed that 501 were consistently altered (P-value < 0.05) in the oviduct in the presence of Y-chromosome-bearing spermatozoa compared to the presence of X-chromosome-bearing spermatozoa. From these 501 transcripts, 271 transcripts (54.1%) were down-regulated and 230 transcripts (45.9%) were up-regulated when the Y- chromosome-bearing spermatozoa was present in the oviduct. Our data showed that local immune responses specific to each sperm type were elicited within the oviduct. In addition, either type of spermatozoa elicits sex-specific signal transduction signalling by oviductal cells.

**Conclusions:**

Our data suggest that the oviduct functions as a biological sensor that screens the spermatozoon, and then responds by modifying the oviductal environment. We hypothesize that there might exist a gender biasing mechanism controlled by the female.

**Electronic supplementary material:**

The online version of this article (doi:10.1186/1471-2164-15-293) contains supplementary material, which is available to authorized users.

## Background

For many years gender allocation of offspring in mammals, including humans, has been regarded as a matter of chance, depending on whether an X- or a Y- chromosome-bearing spermatozoon reaches the oocyte first. Since an equal number of X- and Y- spermatozoa are produced during spermatogenesis [1], and fertilization is a random event, it stands to reason that in each generation equal numbers of males and females should be born. Evidence from the field and laboratory challenges this classic dogma and suggests that some kind of adaptive control of offspring gender may exist in mammals [2, 3]. Evidence for this ability exists in many invertebrates and some avian species are able to adjust their progeny sex ratio predictably in response to environmental conditions [4]. Numerous factors such as population density, resource availability (famine), season, mother’s age, levels of hormones, time of insemination and stress are known to influence the sex ratio in mammals [5–8]. However, the biological mechanism(s) through which mammals can bias the offspring ratio is still unknown.

Several hypothetical mechanisms have been proposed to explain sex ratio skewing in mammals. On the male side, any shift from the expected 1:1 sex ratio among offspring has been related to intrinsic differences in sperm motility, viability and fertilization ability of the two gametes types [9]. On the female side, the condition of the reproductive tract and the penetrability of the oocyte’s zona pellucida, which varies according to the timing of insemination relative to ovulation, have been suggested to influence differentially the ability of X- or Y-spermatozoa to fertilize oocytes [10]. Once fertilization has occurred, the milieu of the oviduct, and subsequently the environment of the uterus, may affect the developmental rates of XX-embryos and XY-embryos [11, 12].

Given that the female investment in the offspring is considerably larger than that of the male, it is more probable that a mechanism to bias the offspring sex ratio is operated by the mother [13]. Furthermore, it is more likely that such a hypothetical mechanism would operate in the oviduct immediately before, or at the time of, fertilization because it is less costly to females than other suggested mechanisms acting later during pregnancy [2, 11]. The mammalian oviduct is the venue for important reproductive events such as sperm and oocyte transport, sperm binding and release, fertilization and early-embryonic development [14]. In addition, the oviduct is implicated in the selection of spermatozoa, being capable of distinguishing between good and poor sperm quality [15].

**Figure 1:**
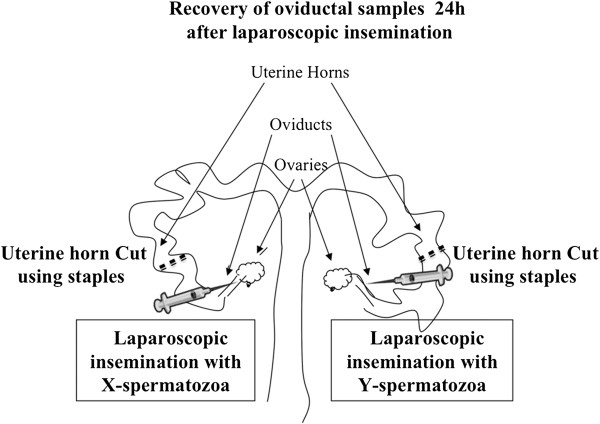
**Schematic representation of the experimental design.** Sows were subjected to laparoscopic surgery. To prevent X- and Y-spermatozoa migration between oviducts, both uterine horns were cut using titanium staples. Then, one oviduct was inseminated with X-spermatozoa and the contralateral oviduct was inseminated with Y-spermatozoa (3 × 10^5^ spermatozoa/100 μl) from the same animal. Twenty-four hours following laparoscopic insemination, oviductal tissues containing X- and Y-sperm samples were collected from each side of the reproductive tract in all animals.

Here, we address the question of whether the female may distinguish between the presence of X and Y-spermatozoa in the oviduct before fertilization occurs. We tested this possibility by examining whether the presence of the X- and Y-spermatozoa elicit different transcriptomic responses within the oviduct. To test our hypothesis we used an *in vivo* pig model that directly compared the oviduct containing Y-spermatozoa to the contralateral oviduct from the same animal, but containing X-spermatozoa (Figure 1). The advantages of this model were: 1) that minimize the confounding factors known to bias the sex ratio [2] since both oviducts analyzed were from the same animal and therefore were under the same nutritional, health and hormonal environment, and 2) that avoid the possibility that oocytes could mask the oviductal responses towards X- and Y-spermatozoa, because only sows showing multiple pre-ovulatory follicles were selected for this study. It has previously been reported that, like spermatozoa, oocytes elicit distinct proteomic alterations [16].

Our study add a complete new layer of competition to the mating game, since up to date most studies of offspring sex ratio are based on epidemiological studies, showing a traditional maternal dominance or lately a male influence in specific species. We open up a new perspective in “the battle of the sexes”, suggesting that this battle starts in the oviduct and providing the first molecular evidence of a sex-specific sperm recognition system in the oviduct.

## Results and discussion

**Figure 2:**
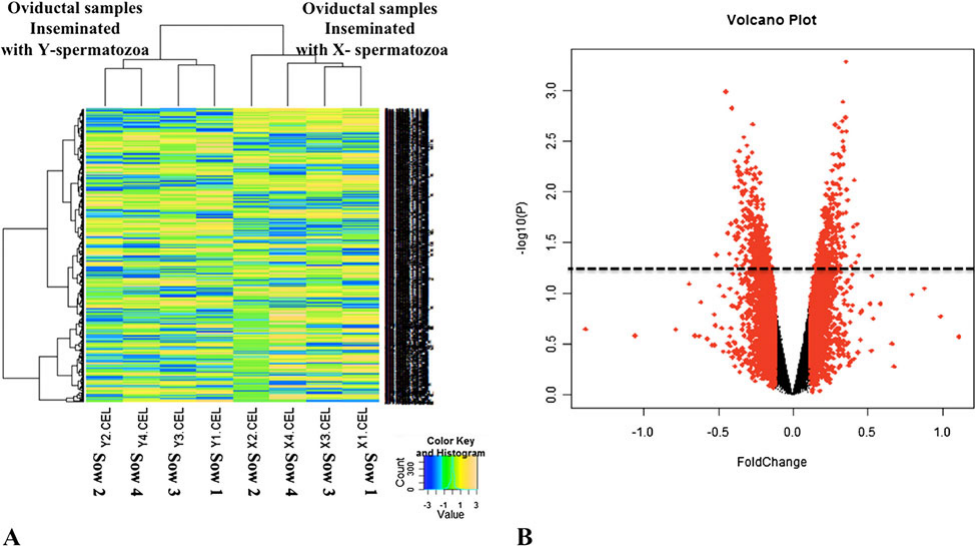
**The presence of Y-spermatozoa elicited different transcriptome response within the oviduct when compared to X-spermatozoa. A**: Cluster heat map analysis of the transcriptional profiles obtained from oviductal samples inseminated with X-spermatozoa and Y-spermatozoa. Each row represents a different gene, and each column displays gene expressions at different samples (X1-X4 for oviductal samples inseminated with X-spermatozoa; Y1-Y2 for oviductal samples inseminated with Y-spermatozoa). Data values displayed as yellow and blue represent elevated and reduced expression, respectively. **B**: A Volcano plot depicting significant changes in gene expression between oviductal samples inseminated with Y-spermatozoa and X-spermatozoa. Each of the 23,124-oligonucleotide probes is represented by a dot in the graph. The x-axis represents the fold change and the y-axis represents the statistical significance (-log10 of p-value). Transcripts showing significant differences in gene expression (501 probes, p < 0.05) are above the broken line.

Our work showed that the presence of X- and Y-spermatozoa did indeed elicit different transcriptomic responses within the oviduct (Figure 2A). Around 2% of transcripts (501 out of 24123 probes from Affymetrix Porcine Chip) were consistently altered (P-value < 0.05) in the oviduct in the presence of Y-chromosome-bearing spermatozoa compared to the presence of X-chromosome-bearing spermatozoa (Figure 2B). From these 501 transcripts, 271 transcripts (54.1%) were down-regulated and 230 transcripts (45.9%) were up-regulated when the Y- chromosome-bearing spermatozoa was present in the oviduct. A complete list of the transcripts altered in the oviduct inseminated with Y- chromosome bearing spermatozoa compared to X -chromosome bearing spermatozoa is presented in the Additional file 1.

To obtain a biologically meaningful overview of the altered transcripts in the presence of Y-chromosome-bearing spermatozoa compared to X-chromosome-bearing spermatozoa, genes differently expressed were organized into different categories and subcategories according to KEGG database hierarchy. The functional categories with higher number of genes were: signal transduction, immune system, digestive system and endocrine system. The pathways in which these altered transcripts were involved with are presented in Table 1. Interestingly, a higher number of genes involved in signal transduction and immune system were up-regulated (60-70%) in the presence of Y-chromosome bearing spermatozoa when compared to X- chromosome-bearing spermatozoa. Other interesting subcategories with high numbers of transcripts involved were: nervous system, cell growth and death, cell communication, signalling molecules and interaction, folding, sorting and degradation and transport and catabolism. The results of all data pathways classification are available in Additional file 2.

**Table 1:** **Pathways and altered genes involved in signal transduction, immune system, digestive system and endocrine system**

KEGG category	KEGG subcategory	KEGG pathways	Transcripts
*Environmental Information Processing*	Signal Transduction	Wnt signaling pathway	PPP2R5C, FBXW11, SMAD4, WNT5B, PRKCA
		MAPK signaling pathway	TGFB1, FGF12, CACNA1B, CRK, FGFR1, PRKCA, MAP4K3, MAP3K11
		TGF-beta signaling pathway	TGFB1, SMAD5, LTBP1, SMAD4, BMP4
		mTOR signaling pathway	ULK2, PRKAA1, HIF1A
		Calcium signaling pathway	PDE1A, CACNA1B, GNAL, HTR2A, ERBB4, ADCY9, PHKA1, PDE1B, PRKCA, NOS2A
		ErbB signaling pathway	CRK, ERBB4, CDKN1B, PRKCA
		Phosphatidylinositol signaling system	DGKI, PRKCA, INPP4B
		Jak-STAT signaling pathway	LEPR, LIF
		Hedgehog signaling pathway	FBXW11, WNT5B, BMP4
		VEGF signaling pathway	PRKCA
*Organismal Systems*	Immune System	Intestinal immune network for IgA production	TGFB1
		Chemokine signaling pathway	CCL8, CRK, ADCY9, VAV2
		Hematopoietic cell lineage	CD55
		Complement and coagulation cascades	CD55, TFPI, C5AR1
		Fc gamma R-mediated phagocytosis	CRK, PPAP2A, PRKCA, VAV2
		Leukocyte transendothelial migration	CTNNA3, CLDN10, MMP9, PRKCA, VAV2
		Natural killer cell mediated cytotoxicity	FCER1G, PRKCA, VAV2
		Fc epsilon RI signaling pathway	FCER1G, BTK, PRKCA, VAV2
		Toll-like receptor signaling pathway	MYD88, IRF7
		B cell receptor signaling pathway	BTK, VAV2
		RIG-I-like receptor signaling pathway	ATG5, IRF7
		Cytosolic DNA-sensing pathway	IRF7
		T cell receptor signaling pathway	VAV2
	Digestive System	Vitamin digestion and absorption	SLC23A1
		Mineral absorption	CYBRD1, ATP1B2, ATP1A2, ATP7A
		Fat digestion and absorption	PPAP2A
		Salivary secretion	ATP1B2, ATP1A2, ADCY9, PRKCA
		Gastric acid secretion	ATP1B2, ATP1A2, SSTR2, ADCY9, PRKCA
		Pancreatic secretion	ATP1B2, ATP1A2, ADCY9, CLCA1, PRKCA
		Bile secretion	ATP1B2, ATP1A2, ABCB11, ADCY9, ABCB1
		Carbohydrate digestion and absorption	ATP1B2, ATP1A2, SLC2A2
		Protein digestion and absorption	ATP1B2, ATP1A2, COL11A1
	*Endocrine System*	Progesterone-mediated oocyte maturation	CDC27, SPDYA, ADCY9, CCNA1
		Insulin signaling pathway	CRK, PCK2, PRKAA1, PHKA1, IRS4, FLOT2
		Adipocytokine signaling pathway	ACSBG1, PCK2, LEPR, ADIPOQ, PRKAA1, IRS4
		PPAR signaling pathway	ACSBG1, PCK2, ADIPOQ, FABP7, PLTP
		GnRH signaling pathway	ADCY9, PRKCA
		Melanogenesis	ADCY9, WNT5B, PRKCA

Our data provide the first evidence to show how spermatozoa carrying the Y- or X-chromosome can modulate the oviductal response by activating specific signalling pathways in a gender specific manner. These data imply that the female reproductive tract recognizes the presence of X- or Y-chromosome-bearing spermatozoa in the oviduct before fertilization occurs. On this basis, we hypothesize that a sex-specific sperm recognition system exists in the female reproductive tract. Furthermore, we propose that this sperm recognition system can modulate the gender selection of the offspring. Our hypothesis challenges two long-held assumptions: 1) on the female side, that the reproductive tract is a passive participant in sperm selection [17] and 2) on the male side, that there are no differences in morphology, metabolic activity or functional abilities of X and Y sperm that could be used by the female tract to recognize the two types of sperm [18].

Recent research has tended to suggest that the female tract is an active participant in the sperm selection process [15, 19]. Furthermore, the oviduct is emerging as a chief protagonist in the sperm selection process, facilitating/or inhibiting sperm transport and allowing only selected sperm to reach the oocyte. This poses the question of whether spermatozoa are pilots or merely passengers in the sperm journey to the oocyte. It is estimated that from the approximately 30 billions of spermatozoa deposited into the female reproductive tract after insemination, only around 1000–5000 spermatozoa enter the oviduct in the pig and bind to the oviductal cell surface [20]. Once in the oviduct the spermatozoa are subjected to several further selection processes before being able to interact with oocytes. This oviductal selection process seems to be based on the intrinsic integrity and information content of the sperm DNA or/and based on more subtle properties that reflect the individual spermatozoon [15]. Thus, we speculate that the oviduct could also differentiate between X and Y-spermatozoa.

Researchers have for many years investigated the differences in size, shape, motility or differentially expressed proteins between X- and Y-spermatozoa [21–23] and no conclusive findings have emerged, aside from the very small 2.8-4% difference in genetic material (depending on species). This small difference in DNA content, due to the arm of the X chromosome that is not present on the Y, is tightly packaged in a semi-crystalline form inside the spermatozoon [24] and does not seem to be accessible for evaluation by external systems in the laboratory. Our new findings open the possibility that the oviductal recognition of X- and Y-spermatozoa may be intimately related to currently unknown differences in morphology or metabolism of X- versus Y-bearing sperm. Recently researchers have identified different topographic characteristics on the head of X- and Y-spermatozoa, viewed on a nanometric scale using atomic force microscopy [25]. Another study has pointed to differentially expressed proteins between bull X- and Y-spermatozoa involved in energy metabolism, stress resistance and cell defence [23]. Chen and colleagues [23] even suggested that there could be differences in the way that energy is produced and varied vulnerabilities to environmental changes between X- and Y-spermatozoa. It is known, for example that different metabolic rate is related to dissimilar production of ROS [26]. Thus, we speculate that the different sperm metabolism identified by Chen and colleagues [23] could lead to the release of distinct amount of ROS substances or diverse ROS metabolites to the oviduct by each type of spermatozoa. To investigate this we measured sperm motility between X and Y-sperm samples (89.94 ± 1.26% and 91.55 ± 0.78%, respectively), sperm viability (89.57 ± 1.3 and 90.57 ± 1.61 respectively) and intracellular ROS generation (124.74 ± 17.96 and 126.89 ± 19.54 FU/10^12^ live spermatozoa, respectively). As our analyses did not reveal any differences we conclude that the oviduct’s ability to differentiate between the two types of spermatozoa must be based on other mechanisms. Nevertheless, our data demonstrated that the specific oviductal response to each type of sperm was not as a result of a higher number of dead sperm in one type of sperm sample compared to the other or a higher percentage of sperm damage by the flow sorting method and therefore a higher production of ROS.

**Figure 3:**
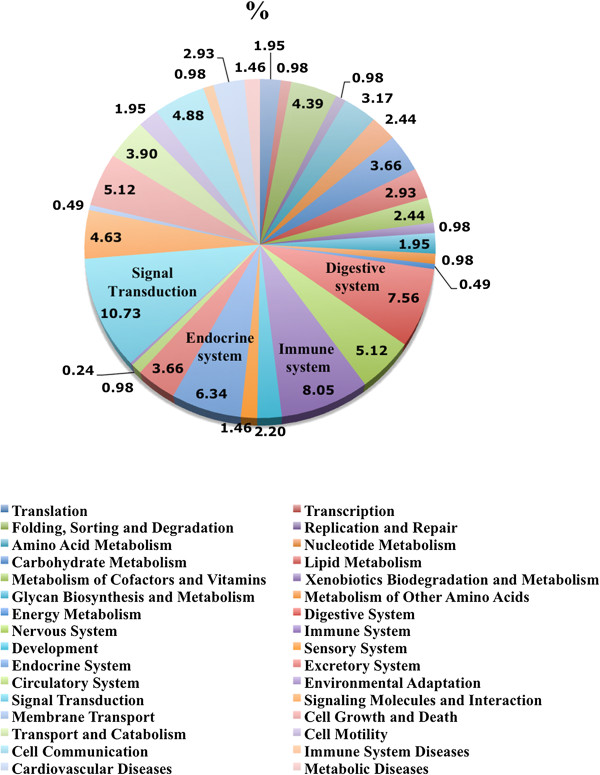
**Transcripts modulated by X-spermatozoa and Y-spermatozoa in the oviduct organized into functional categories.** Transcripts differentially expressed in oviductal samples inseminated with X-spermatozoa compared to Y-spermatozoa organized into functional categories on the basis KEGG PATHWAY database.

**Figure 4:**
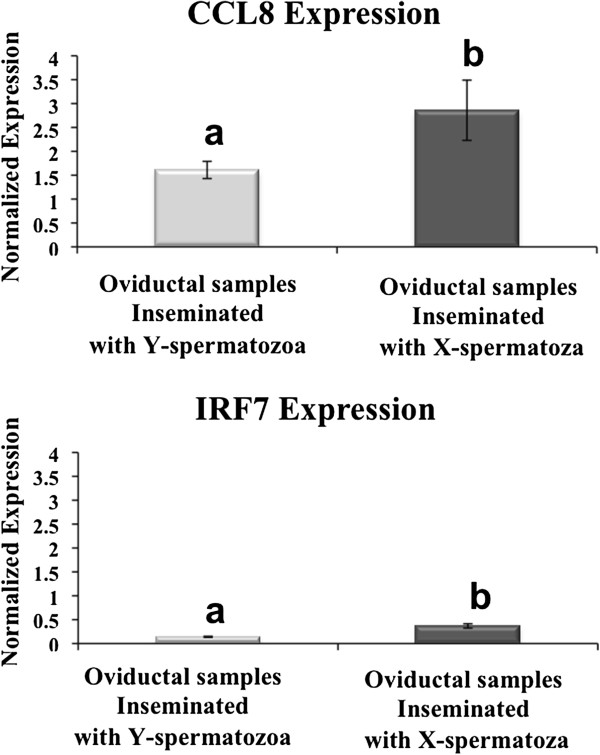
**Validation of the microarray results by qPCR analysis.** CCL8 (chemokine (C-C motif) ligand 8) and IRF7 (interferon regulatory factor 7) expression values (normalized based on ß–actin and Ubiquitin B expression values) in oviductal samples inseminated with X-spermatozoa compared to oviductal samples inseminated with Y-spermatozoa. The expression of both transcripts in the oviductal samples inseminated with Y-spermatozoa was significantly different from that of the oviductal samples inseminated with Y-spermatozoa (P < 0.05).

Immunological strategies to gender selection have also been proposed since the finding of a family of gene products encoded in, or controlled by, the Y chromosome that is only present on male’s cell surfaces [27]. This idea, that sex selection in mammals occurs through a specific immune response, is in line with our results. Bioinformatics analysis of our microarray data showed that genes involved in signal transduction and immune-related genes were the most representative of the altered genes in this study (Figure 3). Moreover, a higher number of genes involved in these pathways were up-regulated (60-70%) in the presence of Y spermatozoa when compared to X spermatozoa (Supplementary data). Particularly interesting was the fact that the immune system seems to be involved in the recognition of X and Y spermatozoa within the oviduct. It is logical to assume that the immune system plays a key role in ensuring tolerance to spermatozoa in the maternal tract. Under normal circumstances, when the maternal tract is exposed to pathogens or a non-self-entity, the immune system responds in an aggressive manner. However the spermatozoon, which is a non-pathogenic “foreign invader” to be destroyed (non-self-entity), is accepted in the maternal tract and is guided to the oocyte. We suggest that the immune system might act as a molecular screening process in the oviduct that allows only preferred X- or Y-spermatozoa to reach the oocyte. Interferon regulatory factor 7 (IRF7) and Chemokine (C-C motif) ligand 8 (CCL8), which are involved in the immune system as signalling molecules were further confirmed using qPCR (Figure 4). IRF7 is associated with specific families of pattern recognition receptors such as Toll-like receptors (TLRs), RIG-I-like receptors (RLRs) and Cytosolic DNA-sensing pathway [28]. All of these pathways are responsible for detecting microbial pathogens or foreign DNA from invading microbes or host cells and may also participate in the recognition between X and Y sperm. The current data further corroborated and confirmed our previous report showing a distinct response of oviduct to spermatozoa of unsorted ejaculates [16]. However, due to the limited sample size, the results presented here should be interpreted with caution. Our future experiments would be directed towards increasing the sample size of the study as well as establishing an *in vitro* based system for understanding the mechanism(s) mediating this process.

## Conclusions

The present investigation demonstrates for the first time, distinct alterations of oviductal gene expression in response to X and Y spermatozoa. These changes imply that the oviduct is able to distinguish between X and Y-spermatozoa and thereby fine-tune its physiology and gene expression in response. We propose that the oviduct functions as a biological sensor that screens the spermatozoa, allowing only a preferred cohort to proceed. Our findings point to the existence of a sex-specific-sperm recognition system in the oviduct that alerts the mother to the presence of the X or Y-spermatozoa, thereby expanding currently proposed hypotheses that influence sex ratio populations.

## Methods

### Experimental design

To determine the alterations in the oviduct transcriptome in response to X and Y sperm our unique experimental model together with Affymetrix microarray technology were used. Figure 1 provides a schematic overview of the experimental model employed. Four biological replicates were performed (n = 4 sows) and a total of 8 arrays were used for microarray study (4 arrays for oviduct samples containing Y- bearing spermatozoa and 4 arrays for oviduct samples containing X-bearing spermatozoa). To validate the microarrays results, two transcripts were selected (Chemokine (C-C motif) ligand 8 (CCL8) and Interferon regulatory factor 7 (IRF7)) and gene expression was analyzed by qPCR. For this experiment, 4 biological replicates (n = 4 sows) and 3 technical replicates were performed.

### Animals

Weaned crossbred sows from Landrace × Large White genetic line (from two to six parities) were selected for these experiments. All experiments were performed after obtaining approval from the Ethical Committee for Experimentation with Animals of the University of Murcia, Spain (385/2008).

### Detection of oestrus and ovarian status

Oestrus detection was carried out once a day, 2 days after weaning, by exposing females to a mature boar and applying manual back-pressure. Females that showed a standing estrous reflex were considered to be in heat and the ovaries were scanned by transrectal ultrasonography [29]. Only sows showing multiple pre-ovulatory follicles (diameter of antrum >6 mm) were selected for experiments. Inseminations were carried out within 2–3 h after the ultrasonography and oviduct samples collection took place 24 h later (approximately 12-24 h before ovulation took place in most of the sows).

### Semen collection and flow cytometric sorting

Sperm-rich fractions from three fertile mature boars, that had previously sired offspring, were collected by gloved-hand method and extended in Beltsville Thawing Solution (BTS) to 150 × 10^6^ spermatozoa/mL [30]. After collection, samples were evaluated for normality (motility >80%, membrane integrity > 85%, total sperm count per ejaculate > 20 × 10^9^; acrosomal abnormalities < 10%, abnormal sperm morphology < 15%) [31].

The extended semen was then processed for sperm sorting following the general procedure as previously described [32]. Briefly, 1 mL of extended semen was stained with Hoechst-33342 fluorophore (0.3 mM per 1 × 106 spermatozoa) and incubated for 1 h in darkness at 35°C. After incubation, samples were passed through a 30 mm nylon mesh filter to remove debris or clumped spermatozoa. Then, 1 μl/ml of food dye (1 mg/ml; Warner Jenkinson Company Inc., St. Louis, MO, USA FD&C 40 solution) was added to the sample to identify dead sperm and kept 15 minutes at room temperature in dark. The stained spermatozoa were separated using a high speed MoFlo SX flow cytometer/sperm sorter (Dako Colorado Inc., Fort Collins, Co, USA), equipped with a solid state laser (ultraviolet wavelength, 351-363 nm) at 175 mW (Spectra Physics 1330, Terra Bella Avenue, Mountain View, CA, USA). Spermatozoa were separated into X- and Y- chromosome bearing populations with 91% purity of Y-spermatozoa and 93% of X-spermatozoa. Both the X sperm and the Y sperm populations were recovered from the three boars. Consequently, in one side the three X-insemination doses were pooled and in the other side the three Y-insemination doses from the three boars were also pooled and both doses finally diluted in BTS to 3 × 10^5^ spermatozoa/100 μl before laparoscopic insemination.

### Sperm motility, viability and functionality of X- and Y-sperm samples

The motility of X- and Y-sperm samples was evaluated objectively using a computer-assisted analysis system (ISAS; Proiser R + D, Paterna, Spain). The sperm motility variable recorded was the overall percentage of motile spermatozoa (average path velocity (VAP) = 20 mm/s) [31].

The viability of the spermatozoa was evaluated by simultaneous cytometric assessment of the plasma and acrosomal membrane integrity by using a triple-fluorescence procedure as described by Martinez-Alborcia et al., [33]. Briefly, a 100 μl sperm sample (30 × 106 cells/ml in PBS) was transferred to culture tubes containing 2.5 μl H-42 (0.05 mg/ml in PBS), 2 μl propidium iodide (PI, 0.5 mg/ml in PBS, Molecular Probes Europe BV, Leiden, The Netherlands) and 2 μl fluorescein-conjugated peanut agglutinin (PNA-FITC, 200 μg/ml in PBS). The samples were mixed and incubated at 38°C in the dark for 10 min. Immediately before analysis by flow cytometry, 400 μl PBS was added to each X- and Y- sperm samples and mixed. The fluorescence spectra of PI and PNA-FITC was detected using a 670 nm long-pass (LP) filter and a 530/30 nm BP filter, respectively. The spermatozoa analyzed were categorized into four categories: (1) intact plasma and acrosomal membranes (PI-/PNA-FITC-); (2) intact plasma membrane and damaged acrosome (PI-/PNA-FITC+); (3) damaged plasma membrane and intact acrosome (PI+/PNA-FITC-); or (4) damaged plasma and acrosomal membranes (PI+/PNA+). The viable spermatozoa exhibited intact plasma and acrosomal membranes and this was expressed as a percentage of the total cells.

The functionality of the spermatozoa was assessed by the intracellular ROS production in X and Y-sperm samples. The intracellular production of ROS by each sperm sample was measured using 5-(and- 6) chloromethyl-20,70-dichlorodihydrofluorescein diacetate acetylester (CM-H2DCFDA), described by Martinez-Alborcia et al., [33].

CM-H2DCFDA is freely permeable across cell membranes and becomes incorporated into the hydrophobic regions of the cell. Upon entering the cell, the acetate moiety of CM-H2DCFDA is cleaved by cellular esterases to leave the impermeant and non-fluorescent molecule 20,70-dichlorodihydro- fluorescein (H2DCF). The H2DCF is oxidized by hydrogen peroxide (H2O2) into dichlorofluorescein (DCF), which fluoresces at 530 nm following excitation at 488 nm. For each X-and Y-sperm sample, two different 50 μl aliquots of mTBM-diluted spermatozoa were diluted in 950 μl PBS containing (1) 1.25 μl H-42 (0.05 mg/ml in PBS), 1 μl PI (1 mg/ml in PBS), 1 μl H2DCFDA (1 mM in DMSO) and 1 μl tert-butylhydrogen peroxide (1 mM in purified water) to induce oxidative stress (first aliquot; used to measure induced ROS formation) or (2) 1.25 μl H-42, 1 μl PI and 1 μl H2DCFDA (second aliquot; used to measure basal ROS formation). The samples were incubated at 38ªC in the dark for 10 min before flow cytometry. The mean fluorescence intensity of DCF (induced minus basal) was expressed as fluorescence units (FU) per 1012 live spermatozoa.

### Intraoviductal laparoscopic insemination

Intraoviductal laparoscopic inseminations were carried out within 2–3 h of the ultrasonography. Sows were sedated by azaperone administration (2 mg/kg body weight, i.m.), anaesthetized with sodium thiopenthal (7 mg/kg body weight, i.v.) and maintained under anesthesia with isofluorane (3.5–5%). Intraoviductal laparoscopic inseminations were carried as described by Almiñana et al., [34]. Briefly, each sow was placed in the supine position and a pneumoperitoneum was established. The abdominal cavity was insufflated with CO2 to 14 mmHg. Two accessory ports were placed in the right and left part of the hemi abdomen, which provided access for laparoscopic Duval forceps for manipulating the uterine horn and grasping the oviduct for the insemination, respectively. To prevent X and Y sperm migration between oviducts, both uterine horns were cut using titanium staples (EndoGIA Universal 60/4.8; Tyco heathcare, Mansfield, MA). To perform the insemination, the oviduct was grasped with the Duval forceps in the isthmus region and the sperm dose containing Y sorted spermatozoa (3 × 105 spermatozoa/100 μl.) was flushed into one oviduct (above of the ampullar region in direction to isthmus). The procedure was then repeated in the contralateral oviduct but injecting the X sperm dose containing the X-chromosome bearing sperm (Figure 1). After both oviducts were inseminated, the trocars were removed and minor suture was required. Following surgery, sows were returned to their accustomed environment.

### Sample collection and RNA preparation

Twenty-four hours following laparoscopic insemination, oviduct tissues containing X and Y sperm populations were collected from each side of the reproductive tract in each animal. Sows were sedated as previously described. To avoid the presence of oocytes that could mask the oviduct response to X and Y sperm, only oviduct samples from sows showing no signs of ovulation were collected. In addition, oviducts were flushed with Phosphate Buffered Saline medium (PBS, 30 ml) and the absence of oocytes was verified by careful examination of oviduct flushings under a stereomicroscope. In cases where it was unsure whether the sow had ovulated oviduct samples were discarded.

Oviducts were opened longitudinally and epithelial cells were isolated by scraping the mucosal epithelial layer with a glass slide. Scraped cells from the uterine horn samples were transferred immediately to Tri Reagent (Sigma, Sigma-Aldrich Co, Madrid, Spain), homogenised, snap-frozen in liquid nitrogen and stored at -80°C until further processing.

Total RNA was isolated using a standard procedure involving phenol:chloroform extraction followed by ethanol precipitation. The quantity (NanoDrop 1000 spectrophotometer) and the quality (Agilent 2100 Bioanalyser; Agilent Technologies) of the RNA samples were analysed. Only samples with satisfactory quality as indicated by the absence of degradation of the ribosomal RNA were used for microarrays and quantitative Real-Time Reverse Transcriptase-Polymerase Chain Reaction (qPCR).

### Microarrays hybridization

Affymetrix Porcine Genome gene expression arrays (Affymetrix, Santa Clara, CA) were used in this study. Total RNA samples were prepared according to the Affymetrix Technical Manual (http://www.affymetrix.com). Briefly, 200 ng of total RNA was converted into cDNA using an oligo(dT) which also carries the binding site for T7 RNA polymerase. Superscript II (Affymetrix) was used to carry out this reaction. After first strand synthesis, residual RNA was degraded by addition of RNaseH and a double-stranded cDNA molecule was generated using DNA Polymerase I and DNA ligase. This double stranded molecule was used as a substrate for the T7 RNA polymerase to produce multiple copies of the cRNA using the Affymetrix IVT labelling system. The cRNA molecules produced incorporated biotin labelled ribonucleotides, which acted as a target for the subsequent detection of hybridization, using fluorescently labelled streptavidin. 13 μg of cRNA molecules were heat fragmented and injected to the Porcine GeneChips in a hybridization solution according to the Affymetrix protocol. Hybridization took place overnight in a rotating hybridization oven at 60 rpm, 45°C for 16 hours. The GeneChip arrays were washed using the Affymetrix Fluidics Station. After washing, the GeneChip arrays were scanned using Affymetrix GC3000 scanner. The resultant images were analysed using the Microarray Suite software version 5.1 (Affymetrix). At the detection level each probe set was designated as present, absent or marginal. Only present transcripts were considered expressed. Microarray experiments were carried out according to MIAME guidelines and the complete experimental data can be obtained online from the NCBI Gene Expression Omnibus (http://www.ncbi.nlm.nih.gov/geo/) submission number GSE47139.

### Microarray data and bioinformatics analysis

Microarrays analysis was performed using Taverna workflow management system (http://www.taverna.org.uk.) [35, 36]. Microarrays data were normalized using Robust Multichip Average (RMA) method [37]. Differentially expressed genes between oviduct inseminated with X spermatozoa and Y spermatozoa were detected through t-tests that were applied to normally distributed data using the Limma R/Bioconductor packages [38] with Benjamini-Hochberg false discovery rate and multiple testing correction to control for Type I errors [39]. From the list of differentially expressed genes, the genes that pass the specified threshold for p-value = 0.05 were selected. Using Affymetrix Porcine Annotation in combination with the improved annotation provided by Tsai et al., [40] for these porcine arrays, differentially expressed transcripts from our microarray data were annotated. To obtain a biologically meaningful overview of the altered transcripts in the presence of Y-chromosome-bearing spermatozoa compared to X-chromosome-bearing spermatozoa, genes differently expressed were organized into different categories and subcategories according to KEGG database hierarchy.

#### Quantitative real-time reverse transcriptase polymerase chain reaction

Gene expression profiles derived from microarray analyses were confirmed using quantitative real-time reverse transcriptase polymerase chain reaction (qPCR). The primers used for qPCR are listed in Table 2. Amplified PCR products were sequenced with forward and reverse primers to verify the resulting product.

**Table 2:** **Primers used for qPCR analysis**

Gene symbol	Affymetrix porcine probe	Primer	Sequence	Product size (pb)
CCL8	*SSc.9957.1.A1_at*	Forward	5′ GCGAGATGGCATTTCTCTCT 3′	119
		Reverse	5′ CACACTTCGGCTTACAAGAGG3′	
IRF7	*SSc.2573.9.1_S1_at*	Forward	5′ GCTGGATGAAGCCAGAACA 3′	97
		Reverse	5′ GGCCCAGGCCTTAAAGAT 3′	
Ubiquitin B	Reference gene	Forward	5′ GTCTGAGGGGTGGCTGCTAA 3′	85
		Reverse	5′ TGGGGCAAATGGCTAGAGTG 3′	
β-actin	Reference gene	Forward	5′ CCTCCCTGGAGAAGAGCTA 3′	131
		Reverse	5′ CTTCATGATGGAGTTGAAGGT 3′	

Total RNA from the oviduct samples (inseminated with Y sperm or inseminated with X sperm) was treated three times with DNase I (DNA-free kit; Ambion.) to remove genomic DNA contamination from samples. First-strand cDNA synthesis was performed using High Capacity cDNA Reverse Transcription Kit (Appied Biosystems). Negative controls were prepared without inclusion of the enzyme (non-reverse transcription controls, RT controls). Reverse transcriptase PCR (RT-PCR) was performed according manufacturer instructions. To evaluate the size of the PCR products 10 μl of each sample was resolved on a 1.2% agarose gel and electrophoresis was performed with 1× TAE buffer and a voltage of 110 V for 40–50 min. The bands were visualized by using an ultraviolet transillumination, and digital images were obtained.

SYBR Green Jump Start (Sigma) master mix (containing 10 μl SYBR Green, 7 μl H2O, 1 μl of forward and reverse primers and 1 μl cDNA) was added to each well of PCR plate and amplification was performed under the following conditions: 40 cycles of 95° for 30 s, 55° for 1 min and 72° for 1 min. Samples without template and RT controls (without the addition of enzyme) for each primer set were included to identify contamination. Triplicate measurements for each group of samples were carried out. Quantitative PCR was performed using Mx3005P QPCR (Stratagene, Waldbronn, Germany). The quantification data were analyzed using MxPro QPCR software version 4.01. Quantitative PCR data were analyzed using the comparative CT method [41]. The results were expressed as mean ± SEM arbitrary gene expression values, normalized on the basis of the two reference expression (ß-actin and Ubiquitin B). Statistical analysis was performed using paired *T*-test to evaluate the significance of difference between expression values of oviduct inseminated with Y sperm versus oviduct inseminated with X sperm (in SPSS, version 14.0 (SPSS Inc., Chicago, IL)). The threshold for significance was set at p < 0.05.

### Availability of additional files

The data set supporting the results of this article is available in the additional files and also is available online from the NCBI Gene Expression Omnibus (http://www.ncbi.nlm.nih.gov/geo/) submission number GSE47139.

## Electronic supplementary material

Additional file 1: **List of candidate transcripts differentially expressed in the oviduct in the presence of Y-bearing spermatozoa compared to X-bearing spermatozoa (P-value < 0.05).** (XLS 152 KB)

Additional file 2: **Transcripts modulated by X and Y-chromosome bearing spermatozoa organized into functional categories according to KEGG database.** (XLS 102 KB)
